# Simple Prediction Model of Axillary Lymph Node Positivity After Analyzing Molecular and Clinical Factors in Early Breast Cancer

**DOI:** 10.1097/MD.0000000000003689

**Published:** 2016-05-20

**Authors:** Mi Joo Chung, Jong Hoon Lee, Sung Hwan Kim, Young Jin Suh, Hyun Joo Choi

**Affiliations:** From the Department of Radiation Oncology (MJC), Kyung Hee University Hospital at Gangdong; Department of Radiation Oncology (JHL, SHK); Department of Surgery (YJS); and Department of Hospital Pathology (HJC), St. Vincent's Hospital, The Catholic University of Korea, College of Medicine, Seoul, Korea.

## Abstract

The aim of this study was to evaluate the association between pretreatment molecular and clinical factors and axillary lymph node metastases in early breast cancer. A total of 367 consecutive breast cancer patients with cT1–2NxM0 who underwent breast conserving surgery and axillary lymph node dissection followed by whole breast irradiation were enrolled. We evaluated the pathologic tumor and node status, tumor differentiation, calcification, and lymphovascular invasion, the status of estrogen receptor (ER), progesterone receptor (PR), epidermal growth factor receptor 1 (EGFR1), and human epidermal growth factor receptor 2 (HER2), the expression of E-cadherin, P53, and Ki-67 index. Totally, 108 (29.4%) of the 367 patients had positive axillary lymph nodes. An increased tumor size (*P* = 0.024), the presence of lymphovascular invasion (*P* < 0.001), and Ki-67 index of >20% (*P* = 0.038) were significantly associated with axillary lymph node metastases on the multivariate analysis. In our study, 86.2% of the patients with all the unfavorable factors had an involvement of axillary nodal metastases, and only 12.2% of the patients with all the favorable predictors had positive axillary nodes. The predictive power was significant on the receiver operating curve (*P* < 0.001). We found that several factors, such as tumor size, lymphovascular invasion, and the Ki-67 index, are independent factors that predict positive ALNM on multivariate analysis for the patients with cT1–2 breast cancer. Clinicians simply could predict the probability of ALNM after verifying the molecular and clinical factors in early breast cancer.

## INTRODUCTION

The status of axillary lymph node metastasis (ALNM) in breast cancer is considered as the most important predictor of post-treatment recurrence and survival.^1^ Although relatively simple to perform, the physical examination of axillary lymph nodes is inaccurate with up to 60% false negativity. Thus, axillary dissection or a sentinel lymph node biopsy is a standard modality for axillary staging and treatment in breast cancer.^2^ The ability to predict ALNM may be useful for physicians to select breast cancer patients who have a low risk of ALNM and avoid a full axillary dissection which is associated with lymphedema, shoulder stiffness, and the loss of sensation of the inner arm.^3^

Breast cancer is a biologically heterogeneous subtype of disease. Recently, molecule-based predictive assays have enhanced the understanding for the genetic characteristics of breast tumors and their prognosis. Eventually clinicians hope that these insights will continue to identify predictors of ALNM and make it easier for personalized treatments. Several factors have been reported as potential predictors of ALNM, including tumor size, the presence of lymphovascular invasion, and a triple-negative subtype of breast cancer.^4^ Currently, numerous studies are paying attention to molecular markers. But, none of the molecular markers, including E-cadherin, P53, and epidermal growth factor receptor (EGFR), have been identified as definite predictors of ALNM in breast cancer.^5–8^ Thus, we assessed whether these molecular and clinical markers could be used to predict positive ALNM in early breast cancer. If we can accurately predict the ALNM, medical practitioners can offer patients with early breast cancer more personalized treatments.

## METHODS AND MATERIALS

### Patients

Between September 2011 and September 2014, 367 consecutive breast cancer patients, staged cT1–2NxM0, were evaluated. They underwent breast-conserving surgery and axillary or sentinel lymph node dissection or a modified radical mastectomy followed by whole breast irradiation at our institution. We reviewed all the patients’ medical records, including radiology, pathology, operation, and radiation. We obtained Institutional Review Board approval before we reviewed these records. Patient characteristics include the patient's age and tumor palpability. Physicians recorded the palpability of axillary lymph nodes through the physical examination before radiographical identification.

### Tumor Characteristics and Molecular Analyses

We evaluated the tumor size, tumor calcification, differentiation, and lymphovascular invasion, the status of estrogen receptor (ER), progesterone receptor (PR), EGFR1, and human epidermal growth factor receptor 2 (HER 2), the expression of E-cadherin, Ki-67, and P53. All of breast cancer specimens were examined by experienced breast pathologists. Paraffin-embedded specimens were measured by immunohistochemistry, using an automated system and antibodies against Ki-67 (1:100, clone MIB-1; Dako, Glostrup, Denmark), E-cadherin (1:800, clone 4A2c7; Zymed, San Francisco, CA), P53 (1:100, clone SP5; Neomarkers, Fremont, CA), EGFR (1:20, MU 207-UC; Biogenex, San Ramon, CA), ER (1:100, clone SP1; Dako, Glostrup, Denmark), PR (1:200, clone PgR 636; Dako), and HER2 (1:100, clone SP3; Thermo, Fremont, CA). The proliferative activity was determined by immunostaining for the Ki-67 antibody. Ki-67 scoring was quantified by counting at least 500 tumor cells with nuclear staining in 3 randomly selected ×100 to 200 high-power fields. The Ki-67 index was expressed as the percentage of positive cells in each case.

Based on an immunohistochemical analysis, positivity for ER, PR, and P53 were defined as nuclear staining of >10% of tumor cells. Signal intensity was scored on a scale representing range 0 to 8.^9^ Immunohistochemical results of EGFR and E-cadherin were evaluated according to extension and intensity of membranous staining in tumor cells. Extension was defined as the positive tumor cell percentage. EGFR was said to have positive staining when extension was 10% or more. Intensity was defined as 0 = no staining; 1+ = faint cytoplasmic staining in > 10% of tumor cells; 2+ = moderate membranous staining in > 10% but ≤ 50% of tumor cells; 3+ = strong membranous staining in >50% of tumor cells.

Positivity for HER2 was defined as HER2 gene amplification using the fluorescence in situ hybridization by the PathVysion HER2/neu probe kit (Vysis Inc., Downers Grove, IL) or scoring >3 using the immunohistochemistry method.

### Statistical Analyses

The chi-square test was used to evaluate the univariate significance of the correlations between axillary lymph node metastasis and several clinical and pathological factors. A logistic regression model was used to evaluate the multivariate analysis. For our statistical analyses, *P* value of < 0.05 was considered statistically significant.

## RESULTS

Table 1 summarizes the baseline patients and tumor characteristics. The median age of patients was 51 years (range 19–83). Of the 367 patients, 273 (74.4%) were in pT1 tumors and 94 patients (25.6%) were in pT2 tumors. A total of 108 patients (29.4 %) out of 367 patients had positive axillary lymph metastases; 62 patients (57.4%) had pT1 tumors, and 46 patients (42.6%) had pT2 tumors. Based on the molecular profile of the breast tumor, 337 (91.8%) out of 367 patients had positive E-cadherin expressions and 114 (31.1%) patients had positive P53 expressions. The median of Ki-i-67 index in all tumors was 20% (range 3–90). Also, 175 (47.7%) out of 367 patients had Ki-67 indexes of >20%.

**TABLE 1:**
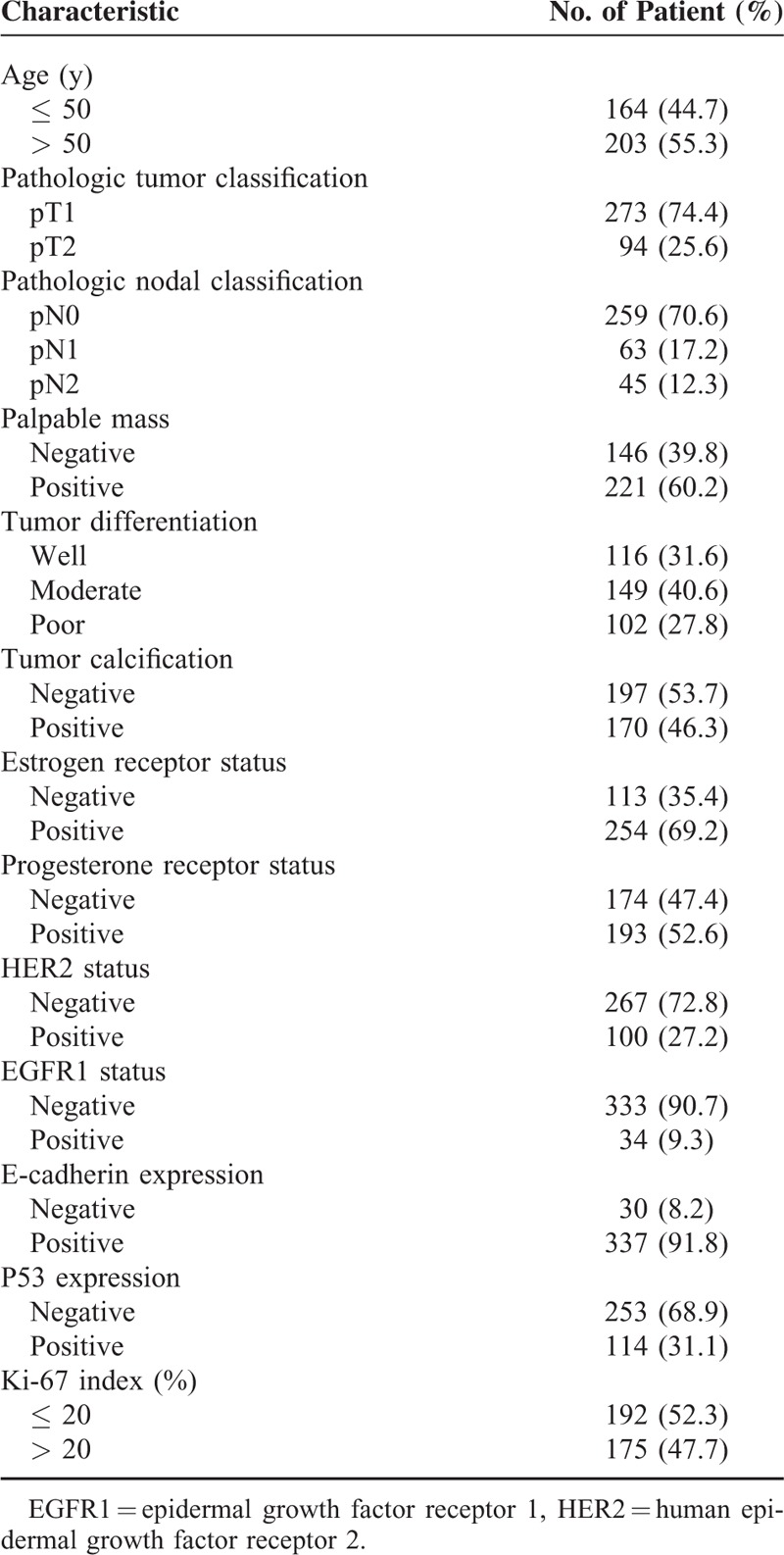
Patient and Tumor Characteristics (n = 367)

**FIGURE 1:**
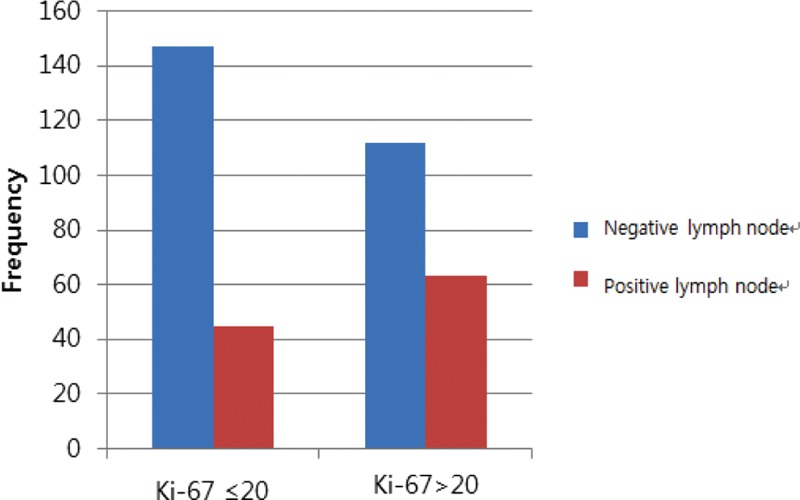
The frequency of nodal positivity according to the Ki-67 index.

Table 2 presents the univariate analysis of factors associated with ALNM. Four factors were correlated with positive ALNM on the univariate analysis. These included an increased tumor size, lymphovascular invasion of the tumor, palpable mass at the time of diagnosis, and a Ki-67 index of > 20%. There was no significant association between the molecular marker of ER, PR, EGFR1, HER2, E-cadherin, P53, and ALNM. A multivariate logistic regression analysis confirmed a significant association between increased tumor size [adjusted odds ratio (OR) and 95% confidence interval (CI), 2.27 (1.42–3.93), *P* = 0.024], the presence of lymphovascular invasion [adjusted OR and 95% CI, 8.43 (5.15–15.29), *P* < 0.001], and a Ki-67 index of >20% [adjusted OR and 95% CI, 1.91 (1.18–2.99), *P* = 0.038] and ALNM. Table 3 provides details of the multivariate analysis. As the level of Ki-67 increased, the frequency of positive axillary nodes significantly increased (Figure 1). Forty-five (41.7%) out of the 192 patients with a Ki-67 index of ≤ 20% had positive ALNM, and 63 (58.3%) of the 175 patients with a Ki-67 index of >20% had positive axillary nodes.

**TABLE 2:**
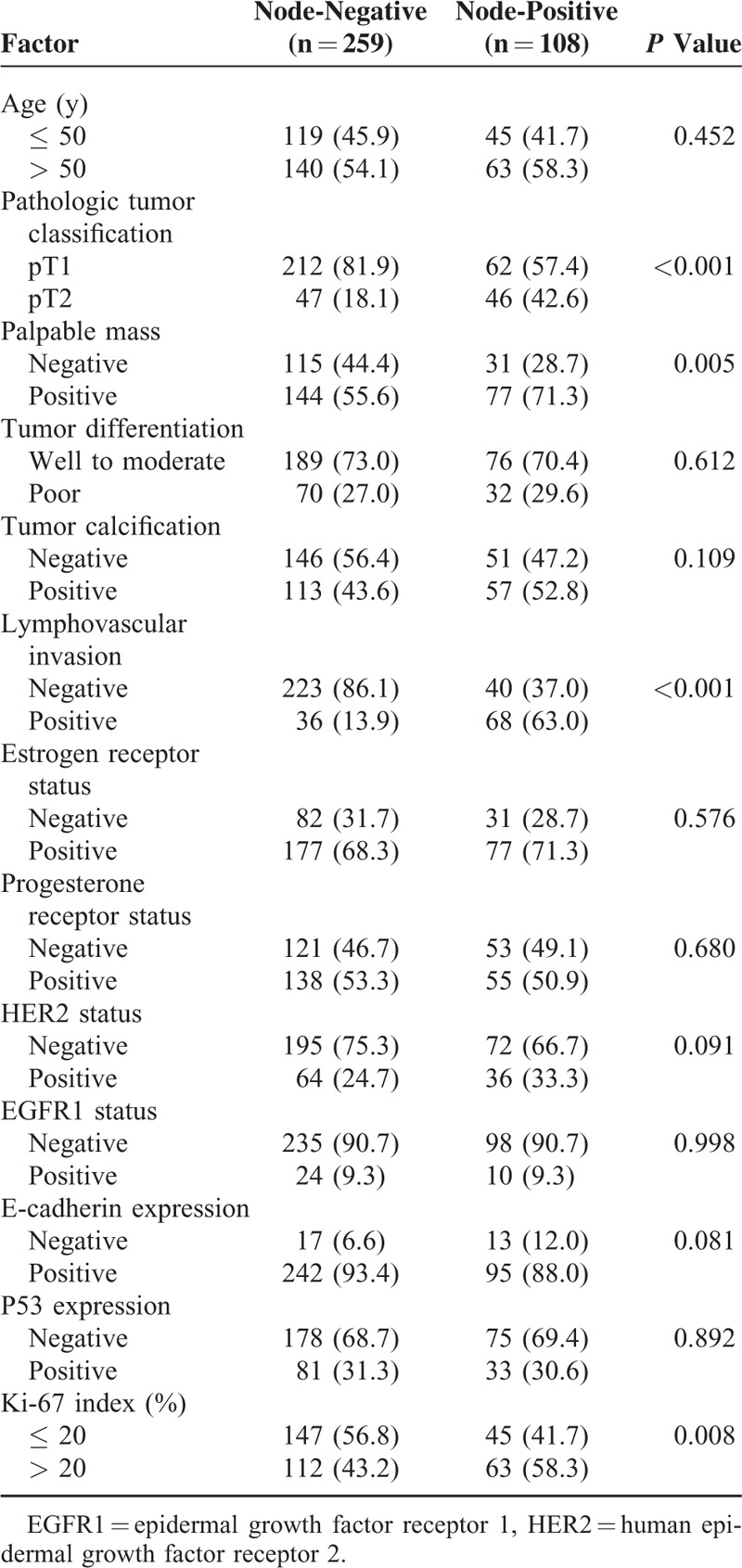
Univariate Analysis of Factors Associated With Axillary Lymph Node Metastasis

**TABLE 3:**
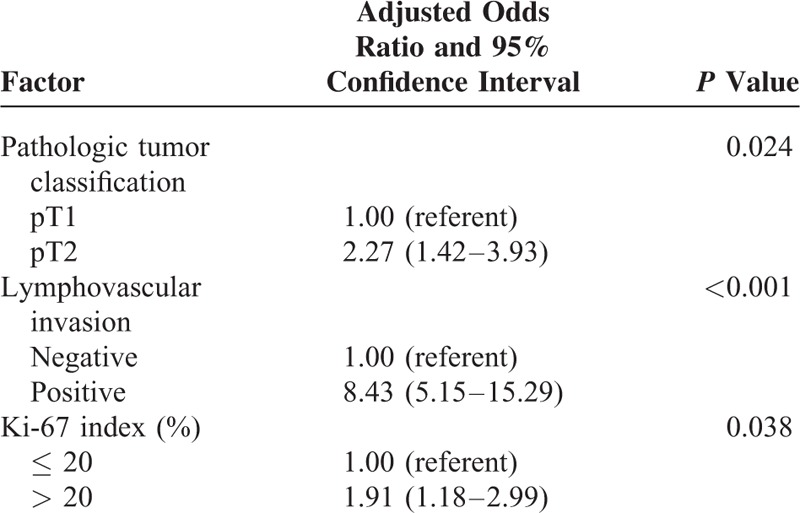
Multivariate Analysis of Factors Associated With Axillary Lymph Node Metastasis

**FIGURE 2:**
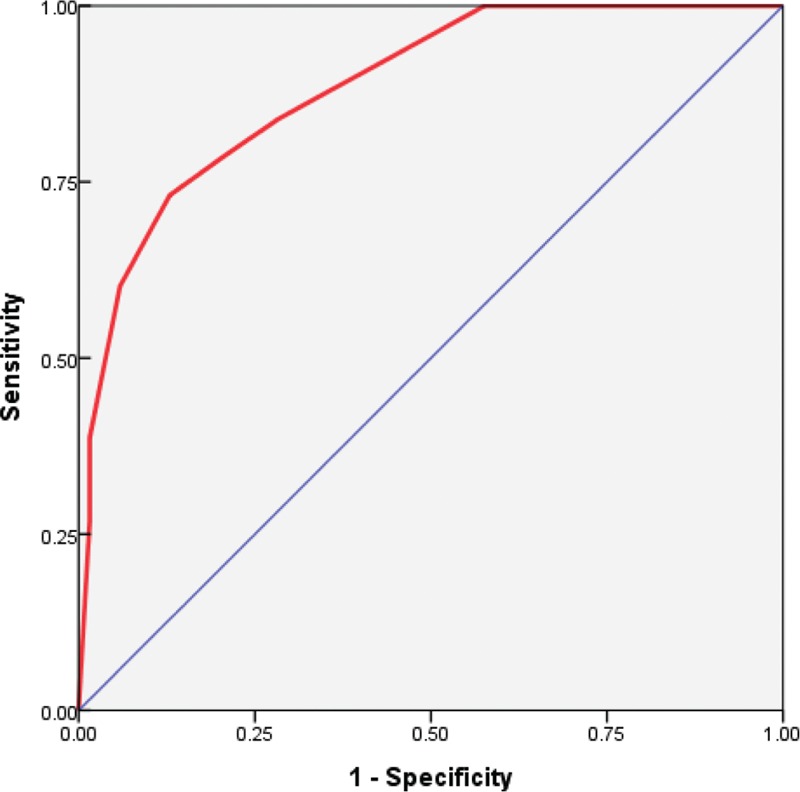
It shows the receiver operating curve (ROC) that corresponds to the multiple logistic model we applied to our data set of 367 patients. The area under the ROC is 0.885 (*P* < 0.001; 95% confidence interval, 0.847–0.922), which indicates the promising predictive power of the multivariate logistic-regression model. ROC = receiver operating curve.

Table 4 lists the proportion of patients who positive lymph nodes for the patients who had some combinations of the potential predictors identified on the multivariate analysis. When all the unfavorable factors such as the presence of lymphovascular invasion, pT2 tumor, and Ki-67 index > 20% were taken into account, a total of 29 patients were identified, and 25 of the 29 patients (86.2%) had an involvement of axillary lymph nodes. On the contrary, when all the favorable factors were taken into account, only 15 (12.2%) of 123 patients had an involvement of axillary lymph nodes. Figure 2 shows the receiver operating curve (ROC) that depicts to the multiple logistic model that was applied to our data set of 367 patients. The area under the ROC curve is 0.885 (95% confidence interval, 0.847–0.922; *P* < 0.001).

**TABLE 4:**
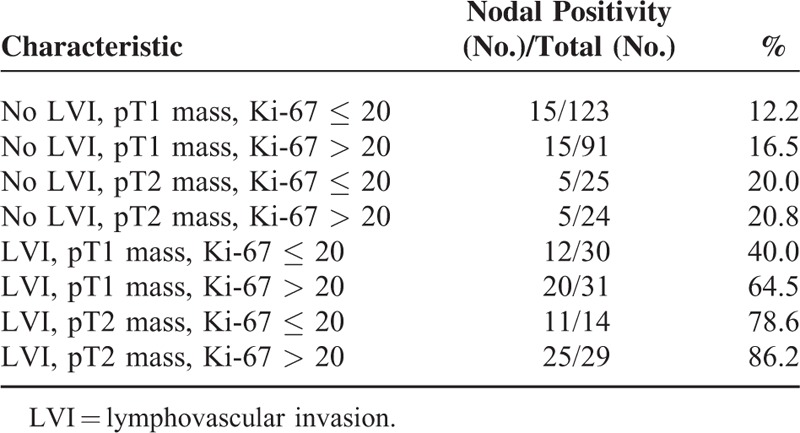
Involvement of Axillary Node According the Combination of Significant Factors Identified on the Multivariate Analysis

## DISCUSSION

The breast has a rich lymphatic plexus; breast tissue typically drains into the axillary lymph nodes. ALNM is an important biological feature of breast cancer, and it leads to poor prognosis and death.^1^ Therefore, axillary lymph node dissection is performed with standard breast conserving surgery even though there is a risk of surgical complications, such as lymph edema and arm dysesthesia.^10,11^ The ability to predict ALNM may be useful for surgeons so that they can modify the axillary treatment plan. The identification of molecular markers that determine the behavior of individual tumors may allow doctors to prioritize patients at different risk levels of developing ALNM.

**FIGURE 3:**
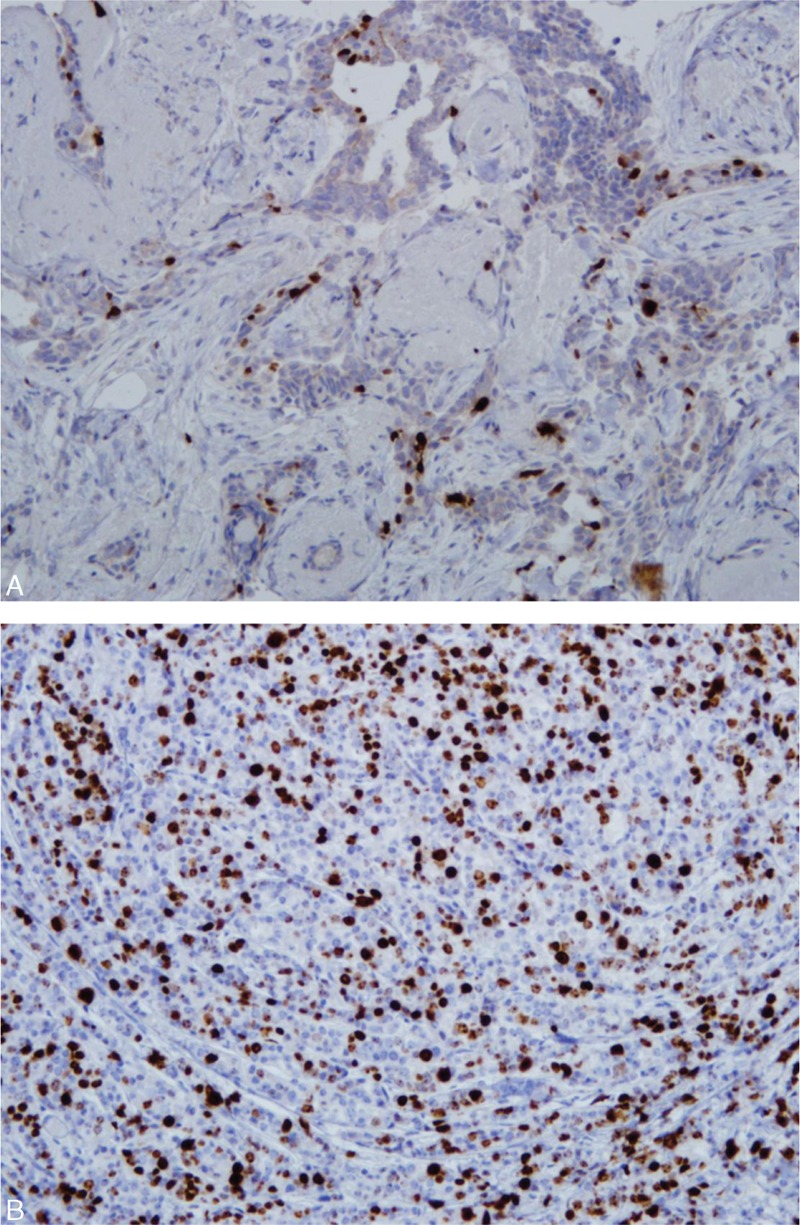
(A) It shows low Ki-67 labeling for breast tumor. (B) Value of >20% on the Ki-67 index for a breast cancer patient with aggressive spreading tumor. (immunohistochemistry, original magnification ×200).

Our aim was to evaluate which molecular markers might be associated with the significant risk of ALNM in early breast cancer. This study mainly focused on classical molecular markers, such as Ki-67, E-cadherin, P53, EGFR, and HER2. Ki-67 is a nuclear protein that is associated with cellular proliferation. In breast cancer, Ki-67 is often correlated with a poor prognosis. The high index of Ki-67 for predicting ALNM is now routinely examined by immunohistochemistry (Figure 3). Yin et al suggested that the level of Ki-67 had a potential value in the prediction of ALNM in invasive breast cancer. In our multivariate analysis, we found that the frequency of ALNM was higher in patients with a Ki-67 index of > 20% than in patients with a Ki-67 index of ≤ 20 (OR, 1.91 and 95% CI, 1.18 to 2.99, *P* = 0.038). The cut-off value of Ki-67 of this study was set at a median value of the Ki-67 index. In several studies, patients were categorized into 2 categories of above and below 20% for the Ki-67 index. The higher Ki-67 index results were significantly associated with ALNM in breast cancer.^5–7^

E-cadherin was mainly localized in the membrane, and faint diffuse cytoplasmic expression was observed. E-cadherin consists of a large extracellular domain composed of smaller transmembrane and cytoplasmic domains and 5 repeat domains.^12^ Overexpression of E-cadherin has often occurred in a number of human epithelial cancers.^13^ E-cadherin genes have been proven to be involved in oncogenesis and cancer development.^14–16^ A few studies had investigated the role of E-cadherin for lymph node metastasis in breast cancer, but the results were not consistent. In our study, there was no significant association between ALNM and E-cadherin expression (*P* = 0.081). Recently, Asiaf et al reported that abnormal E-cadherin methylation occurs in high frequencies in infiltrating breast cancers associated with a decrease in E-cadherin expression. The study found significant differences in tumor-related E-cadherin gene methylation patterns relevant to nodal involvement.^17^ Therefore, a study of abnormal E-cadherin methylation would be necessary in future.

The P53 protein plays a key role for apoptosis in response to DNA damage, P53 overexpression in breast cancer induced poor response to endocrine therapy and chemotherapy.^18–20^ Thus, loss of P53 function is correlated with a high risk of recurrence and death. However, Radha et al reported an equivocal P53 status in both the positive and negative cases of lymph node metastases in immunohistochemistry results.^21^ In this study, the odds ratio of P53 positivity were 0.97 times (95% CI, 0.59–1.57, *P* = 0.892) in people with positive lymph node status. In other words, no correlations were possible between P53 and axillary lymph node metastases.

EGFR is the cell surface receptor for members of the epidermal growth factor family of extracellular protein ligands and plays a role in the regulation of cell proliferation and differentiation.^22^ The EGFR1 is a member of the ErbB family of receptors, a subfamily of 4 closely related receptor tyrosine kinases: EGFR (ErbB-1), HER2 (ErbB2), HER3 (ErbB-3), and HER4 (ErbB-4). HER2 overexpression is reported in about 15% to 20% of breast cancer patients.^23^ A positive HER2 status was related to a positive ALNM.^24^ HER3 overexpression is seen in about 80% of primary colorectal cancer cases and this corresponds to lymph node metastasis.^25^ However, no previous studies have analyzed the molecular status of ALNM and mutations in the EGFR in patients with breast cancer. Therefore, we examined whether it has an association between EGFR and breast cancer with ALNM risk or not. However, in our study, there were no statistical correlations between ALNM and EGFR1 and HER2.

We acknowledge that our series had a number of limitations. First, our study should be understood in view of the inherent biases of a retrospective study design.^26^ Second, we evaluated pT1–2NxM0 patients, not all breast cancer patients.^27^ Thus, our cohorts do not represent all breast cancer patients and need an external validation for the integrity. However, we enrolled 367 consecutive breast cancer patients with pT1–2NxM0 and evaluated them for several molecular markers to assess the exact association between molecular markers and clinical factors and the presence of ALNM. Breast cancer antigen such as CA 15–3 and CA 27.29 are tumor markers widely used for assessing the prognosis of breast cancer patients.^28^ Multiple studies have shown that an increased concentration of CA 15–3 and CA 27.29 are independent predictors of ALNM in patients with early breast cancer who underwent selective lymph node dissection.^29^ However, in our study, we did not examine whether elevation of CA 15–3 and CA27.29 reflected high incidence of ALNM or not.

In conclusion, we found that several factors, such as tumor size, lymphovascular invasion, and the Ki-67 index, are independent factors that predict positive ALNM on multivariate analysis for the patients with pT1–2 breast cancer. The probability of ALNM after verifying the molecular and clinical factors can be simply predicted in early breast cancer.
